# Executives functions in co-occuring adult attention deficit hyperactivity disorder and alcohol use disorder

**DOI:** 10.1192/j.eurpsy.2021.652

**Published:** 2021-08-13

**Authors:** A. D’Alessandro, P. Bendimerad

**Affiliations:** 1 Psychiatry Department, University of Poitiers, Poitiers, France; 2 Psychiatry Department, La Rochelle Hospital, La Rochelle, France

**Keywords:** attention deficit hyperactivity disorder, alcohol use disorder, Executives functions, quality of life

## Abstract

**Introduction:**

Executives functions (EF) are cognitive processes mediating the ability to successfully regulate thoughts and behaviours in order to fulfil a goal. EF impairment has been found both in the Attention Deficit Hyperactivity Disorder (ADHD) and in the Alcohol Use Disorder (AUD). Deficits in EF may have a major impact on patients’ everyday life.

**Objectives:**

The aim of this study was to evaluate EF in a population with a dual diagnosis of Adult ADHD et AUD. We also evaluated the correlation between EF and the dimensions of quality of life.

**Methods:**

For this observational multicentric study, we included patients with AUD starting long-term residential treatment. We used ASRS v.1.1 to screen for Adult ADHD and DIVA to confirm the diagnosis. We assess EF with BRIEF-A. WHOQoL-BREF was used to evaluate quality of life.

**Results:**

Our population consists of 49 patients. Adult ADHD prevalence was 24,49%. Impairment score of all EF explored (Inhibition, Shifting, Emotional Control, Self-Monitoring, Initiative, Working Memory, Planning/Organizing, Organization of materials, Task Monitoring) was higher in patients with co-occuring Adult ADHD and AUD than in patients without Adult ADHD (p<0,001). We found strong negative correlation between Psychological Health and impairment score of Inhibition (p<0,001), Emotion Control (p<0,001), Self-Monitoring (p<0,001) and planning/Organizing (p<0,001). The other dimensions of quality of life were poorly correlated to EF impairment.

**Conclusions:**

The prevalence of Adult ADHD in AUD patients is high. When AUD and Adult ADHD coexist, EF impairment is stronger and quality of life is poorer. Psychological health and EF impairment are strongly associated.

